# Dimensionality and Measurement Invariance of the State-Trait Inventory for Cognitive and Somatic Anxiety (STICSA) and Validity Comparison With Measures of Negative Emotionality

**DOI:** 10.3389/fpsyg.2021.644889

**Published:** 2021-06-07

**Authors:** Isabeau K. Tindall, Guy J. Curtis, Vance Locke

**Affiliations:** ^1^School of Psychological Science, The University of Western Australia, Perth, WA, Australia; ^2^Discipline of Psychology, Murdoch University, Perth, WA, Australia

**Keywords:** STICSA, STAI, anxiety, depression, positive affect, somatic anxiety

## Abstract

This study explored the factor structure of the State-Trait Inventory for Cognitive and Somatic Anxiety (STICSA) and measurement invariance between genders. We also measured concurrent and divergent validity of the STICSA as compared to the State-Trait Anxiety Inventory (STAI). A sample of 1064 (*N* Females = 855) participants completed questionnaires, including measures of anxiety, depression, stress, positive and negative affect. Confirmatory factor analyses supported the original factor structure of the STICSA, which was invariant between genders. Overall, the STICSA had superior concurrent and divergent validity as compared to the STAI. The somatic subscales were also significantly less correlated with depression, and positive and negative affect. Further, the somatic, as compared to cognitive anxiety STICSA subscales were less correlated with depression. This suggests that the STICSA, especially the somatic anxiety subscales, might hold the key to distinguishing between different types of anxiety, as well as between anxiety and depression.

## Introduction

Anxiety can be broadly defined as the anticipation of future threat or danger (American Psychiatric Association, 2013), and it is also considered to be a multidimensional concept (Spielberger,1985a,b; Ree et al., 2008). It has long been established that anxiety can be separated into the dimensions of state anxiety: the transient anxiety response; and trait anxiety: the stable tendency to become state anxious (Spielberger,1985a,b; Spielberger and Sydeman, 1994; Endler and Kocovski, 2001; Kocovski et al., 2004). Traditionally, state and trait anxiety have been measured by the State-Trait Anxiety Inventory (STAI; Spielberger et al., 1983). The STAI has been extensively used in research, cross-culturally (i.e., Spielberger, 1989; Iwata et al., 1998; Spielberger, 2006), within different populations (i.e., adults: Spielberger et al., 1970; Spielberger et al., 1983; adolescents: Rodrigues et al., 2018), and has sound internal consistency and test–retest reliability (Barnes et al., 2002).

Despite the generally positive psychometric features of the STAI, the STAI has been criticized for being unable to adequately distinguish between anxiety and depression (see Grös et al., 2007, 2010). Furthermore, the integrity of the trait anxiety scale has been questioned, as it loads onto negative affect as a higher order factor (Bieling et al., 1998; Bados et al., 2010; Balsamo et al., 2013). The trait scale has also been found to correlate strongly with positive affect, where a lack of positive affect is exclusive to depression, and not to anxiety (Caci et al., 2003). Recent research has also highlighted the inadequacy of the STAI by emphasizing that the STAI does not measure the physical and bodily symptoms of anxiety (Ree et al., 2008; Roberts et al., 2016). Criticisms of the STAI like those above, have led researchers to suggest that the STAI is an inadequate measure of anxiety, and that a better, purer, measure of anxiety is needed (Grös et al., 2007).

Aiming to address the limitations of the STAI, Ree et al. (2008) developed the State-Trait Inventory for Cognitive and Somatic Anxiety (STICSA). In addition to distinguishing between state and trait anxiety, this measure also divides anxiety into the subdomains of cognitive and somatic anxiety (Grös et al., 2007; Ree et al., 2008). Cognitive anxiety is specific to anxiety symptoms related to thought processes, such as worry, and inability to concentrate, while somatic anxiety relates to physiological anxiety symptoms such as hyperventilation, trembling, and palpitations (Ree et al., 2008; Roberts et al., 2016).

The STICSA, due to the division into cognitive and somatic anxiety, in addition to state and trait anxiety, is argued to be superior to the STAI (Grös et al., 2007; Roberts et al., 2016). Like the STAI, the STICSA includes items capturing cognitive anxiety. Unlike the STAI, however, the STICSA also captures variance related to somatic anxiety (Roberts et al., 2016). It is important to examine both cognitive and somatic anxiety, as an individual’s global anxiety score, captured by the STAI, is too broad to allow for determining the separate domains of anxiety (Schwartz et al., 1978; Koksal and Power, 1990; Endler and Kocovski, 2001). For instance, two individuals with the same global trait anxiety score may display quite different profiles of cognitive and somatic trait anxiety (Ree et al., 2008). One individual might exhibit low somatic trait anxiety, but high cognitive trait anxiety, while the other might exhibit the opposite profile. When both individuals are then exposed to a cognitive anxiety induction, the individual with high cognitive trait anxiety may become more state anxious than the individual with low cognitive trait anxiety (Ree et al., 2008). The difference in anxiety profile, therefore, has implications for an individual’s subjective anxiety response, the type of anxiety induction to which they are more likely to become state anxious, and subsequently, their response to specific therapeutic interventions (Koksal et al., 1991).

Due to the potential utility of the STICSA, and conflicting support for different factor structures, it is important to examine the psychometrics of the STICSA. Ree et al. (2008), who originally created the STICSA, through testing an Australian university sample, indicated that the STICSA consisted of two separate factors, one related to state anxiety, subdivided into cognitive and somatic anxiety, and another, related to trait anxiety, also divided into these subdomains (see Supplementary Figure 1). Subsequent researchers have argued against the original factor structure put forward by Ree et al. (2008) and have stated that the STICSA could consist of either a two-factor state-trait model (see Supplementary Figure 2), two-factor cognitive-somatic model (see Supplementary Figure 3) or four-factor state-trait cognitive-somatic model (Grös et al., 2007; see Supplementary Figure 4).

Using a clinical sample, Grös et al. (2007) tested these models and found support for the four-factor state-trait cognitive-somatic model. Replicating this, Balsamo et al. (2015) and Roberts et al. (2016) indicated support for this model when using an older adult Italian sample and Canadian university students, respectively. In extension of Grös et al. (2007) and Balsamo et al. (2015), however, Roberts et al. (2016) also put forward a hierarchical model with a higher order global anxiety factor, and second order intercorrelated state-trait, cognitive-somatic anxiety subdomains (see Supplementary Figure 5). In contrast to these studies (Grös et al., 2007; Balsamo et al., 2015; Roberts et al., 2016), Lancaster et al. (2015) did not find support for the four-factor state-trait cognitive-somatic model while testing African American and European American university students. This study, however, did not compare the factor structure of the alternative models. Most recently, extending on Lancaster et al. (2015), the study by Carlucci et al. (2018) examined the factor structure of all five proposed models using a sample of Italian adults. Contrary to the findings of Grös et al. (2007), Balsamo et al. (2015), and Roberts et al.(2016, but in line with Ree et al. (2008), Carlucci et al. (2018) found evidence for the STICSA when subdivided into separate state (with cognitive and somatic subdomains) and trait (with cognitive and somatic subdomains) anxiety scales. The model proposed by Ree et al. (2008) was also endorsed by Styck et al. (2020) when examining a sample of university students from a Hispanic-serving institution. As seen above, research investigating the internal factor structure of the STICSA is inconsistent (Grös et al., 2007; Ree et al., 2008; Balsamo et al., 2015; Lancaster et al., 2015; Roberts et al., 2016; Carlucci et al., 2018; Styck et al., 2020). Further, to date, limited studies have tried to replicate the factor structure of the hierarchical model (Carlucci et al., 2018; Styck et al., 2020), with this model not supported.

Another caveat with research examining the factor structure of the STICSA has been the use of ethnographically diverse samples and different language versions of the STICSA. Although the STICSA was originally examined using an Australian non-clinical university sample, the STICSA has been examined using Italian (Balsamo et al., 2015; Carlucci et al., 2018), African American (Lancaster et al., 2015) and majority Hispanic (Styck et al., 2020) participants of varying ages. Moreover, the investigation by Grös et al. (2007) tested a clinical sample and so cannot be generalized to a non-clinical population.

As with the factor structure of the STICSA, limited research has examined the concurrent and divergent validity of the STICSA with other measures of anxiety and depression (Grös et al., 2007; Roberts et al., 2016; Carlucci et al., 2018). Despite several studies supporting the superior concurrent validity of the STICSA with measures of anxiety, and divergent validity with depression, as compared to the STAI (Grös et al., 2007, 2010; Roberts et al., 2016; Carlucci et al., 2018), this support is not universal. In the study by Balsamo et al. (2015) the correlation between the cognitive subscales of the STICSA and measures of depression were higher than the correlation of two depression measures with each other. Further, in the study conducted by Lancaster et al. (2015), all subscales of the STICSA were found to be more correlated with measures of depression than with measures of anxiety. These studies called into question the divergent validity of the STICSA, and suggest, as with the STAI, that the STICSA is unable to sufficiently discriminate from depression (Williams et al., 2004; Lancaster et al., 2015). Results of these studies (Balsamo et al., 2015; Lancaster et al., 2015), however, could be specific to the population (older Italian sample) and research questions tested (African American as compared to European American participants). Therefore, future research needs to examine the relationship between the STICSA and anxiety and depression, when using a more representative sample. In addition to examining the divergent validity of the STICSA with depression, it is also important to examine the divergent validity of the STICSA with measures of negative and positive affect (Roberts et al., 2016). Only one study to date has examined negative and positive affect, with this study finding that while the STICSA was similarly correlated with negative affect, the STICSA was less correlated with positive affect as compared to the STAI (Roberts et al., 2016). Therefore, future research also needs to example the relationship between the STICSA as compared to the STAI in relation to affect.

Another area that warrants examination is the influence of gender on the factor structure of the STICSA as well as concurrent and divergent validity. Only one previous study has examined whether the factor structure of the STICSA is invariant across genders (Carlucci et al., 2018). Further, no studies have examined how gender affects the expression of anxiety and subsequently the concurrent and divergent validity of the STICSA with measures of anxiety, depression, and negative and positive affect. Extensive research has suggested that women experience negative emotionally, such as anxiety, to a larger extent than men (Bishop, 1984; Kroenke and Spitzer, 1998; McLean and Anderson, 2009; McLean et al., 2011; Christiansen, 2015). Research has also indicated that gender dictates the expression of anxiety, with women found to suffer from somatic anxiety symptoms, such as faintness and shortness of breath, to a greater degree than men (Sheikh et al., 2002). Balsamo et al. (2015) mentioned that women in their study had significantly higher scores on both subscales of the STICSA state and trait, however, this finding was not explored.

The current study, therefore, sought to further validate the STICSA and its subscales, and to compare the STICSA with the STAI while examining the influence of gender. Specifically, this study aimed to (a) compare and contrast the five different proposed factor models of the STICSA, (b) investigate the concurrent and divergent validity of the STICSA, (c) compare and contrast the concurrent and divergent validity of the STICSA and STAI, and (d) examine these aims while separating between genders.

The STICSA and STAI were compared in terms of concurrent validity with measures of anxiety, that is, the anxiety subscale of the Depression Anxiety Stress Scale (DASS; Lovibond and Lovibond, 1995), and the Anxiety Sensitivity Index (ASI; Reiss et al., 1986). Divergent validity with measures of depression were compared through examining correlations with the Depression Subscale of the DASS (Lovibond and Lovibond, 1995) and the Beck Depression Inventory-II (BDI-II; Beck et al., 1996). Further, divergent validity with measures of positive and negative affect were measured through looking at relationships with the Positive and Negative Affect Schedule (PANAS; Watson et al., 1988). Measures used to examine the concurrent and divergent validity of the STICSA as compared to the STAI, were chosen to allow comparison with previous research comparing the validity of the STICSA and STAI (Grös et al., 2007; Roberts et al., 2016; Carlucci et al., 2018).

Divergent validity with stress, as measured through the stress subscale of the DASS, was also considered (Lovibond and Lovibond, 1995). Although previous research has not examined this comparison, research has indicated that anxiety and stress are theoretically distinct (Lovibond and Lovibond, 1995). Specifically, stress relates to the response elicited by having insufficient resources needed to cope with a situation and is normally proceeded by a distinct event (Sarason, 1984; Lovibond and Lovibond, 1995). While anxiety is specific to the anticipation of a future threat, and therefore, not normally situationally based (Sarason, 1984; Lovibond and Lovibond, 1995).

Due to the exploratory nature of research examining the factor structure of the STICSA, a lack of consensus in the literature, and limited research examining the measurement invariance of the STICSA between genders, no predictions were made as to which model would be the most supported by the sample data. In terms of the concurrent and divergent validity of the STICSA, we proposed two main hypotheses. Firstly, it was hypothesized that the STICSA would be strongly correlated with measures of anxiety, with a stronger correlation expected between the STICSA and these measures as compared to the STAI. This hypothesis was informed by previous research (Grös et al., 2007; Grös et al., 2010; Roberts et al., 2016; Carlucci et al., 2018) and the tripartite model of anxiety and depression (Clark and Watson, 1991). According to the tripartite model (Clark and Watson, 1991), anxiety uniquely loads onto a somatic anxiety/tension factor. Therefore, because of the inclusion of items capturing somatic anxiety within the STICSA, the total scores of both the STICSA state and trait, and the somatic anxiety subscales, were expected to be more correlated with measures of anxiety than the STAI (Grös et al., 2007; Roberts et al., 2016).

Secondly, it was hypothesized that the STICSA would be less correlated with measures of depression, stress, negative and positive affect as compared to the STAI (Clark and Watson, 1991; Grös et al., 2007; Grös et al., 2010; Roberts et al., 2016; Carlucci et al., 2018). The somatic subscale of the STICSA was also predicted to correlate less with depression than the cognitive subscale through this subscale sharing less common variance with depression (Roberts et al., 2016). Further, the STICSA was also predicted to be less correlated with positive affect, as a lack of positive affect is exclusive to depression (Clark and Watson, 1991). The STAI, unlike the STICSA, however, captures variance related to a lack of positive affect (Caci et al., 2003) and so was expected to correlate with positive affect to a larger degree.

## Materials and Methods

According to Simmons et al. (2012), “we report how we determined our sample size, all data exclusions, all manipulations, and all measures in the study” (p. 1).

### Participants

Based on estimates from previous studies, a minimum sample size of 200 participants was required for both male and female samples when conducting confirmatory factor analyses (Kline, 2016). Data was collected according to pre-screening for another study between March 2017 to November 2020, and subsequently, data collection continued until the sample size for that study was reached. A total of 1924 university students and people from the wider community took part in this study. Five participants were under 18, four did not give consent for their data to be used, 365 participants did not fully complete all surveys, and 451 duplicate responses were excluded. The number of duplicate responses is attributable to combining two testing timepoints from the same university. Further, two random responders were removed through examination of scores on scales with reverse scored items and corroborated via examination of consecutive strings of identical responses on other scales. Thus, 1097 participants (females: *N* = 882, females: *M*_*age*_ = 24.54, *SD* = 8.73, males: *M*_*age*_ = 24.88, *SD* = 8.15; total sample: *M*_*age*_ = 24.61, *SD* = 8.61, total sample range: 18–64, 78.9% Caucasian, 11.8% Asian, 5.3% Other Ethnicity, 2.3% African, 1.0% Aboriginal/Torres Strait Islander, 0.7% Hispanic, Latino or of Spanish origin) from Murdoch University took part in this study for partial course credit or the chance to win a gift card. Ethics approval was acquired from Murdoch University before data collection.

### Measures

The State-Trait Inventory for Cognitive and Somatic Anxiety (STICSA; Ree et al., 2008). The STICSA is a self-report questionnaire consisting of 42-items, with 10 items measuring cognitive anxiety and 11 items measuring somatic components of anxiety at both the state and trait level (Ree et al., 2008). An example item for the cognitive trait scale is “I keep busy to avoid uncomfortable thoughts.” Items are rated on a 4-point Likert scale from 1 *(Almost never or Not at all)* to 4 *(Almost always or Very much so)* on the trait and state scales, respectively. Items on the trait scale are asked in terms of “how often, in general, the statement is true of you,” while state items were answered in terms of “how you feel right now, at this very moment.” Higher scores indicate higher levels of anxiety. The STICSA and its subscales have demonstrated good internal consistency and reliability in different populations (e.g., wider community and university students) within previous studies (trait somatic, αs ≥ 0.78, trait cognitive, αs ≥ 0.75, state somatic, αs ≥ 0.75, state cognitive, αs ≥ 0.84; Grös et al., 2007, 2010; Ree et al., 2008; Roberts et al., 2016).

State-Trait Anxiety Inventory (STAI; Spielberger et al., 1970; Spielberger et al., 1983). The STAI is a self-report questionnaire with 40 items rated on a 4-point Likert scale from 1 *(Almost never or Not at all)* to 4 *(Almost always or Very much so)* on the trait and state scale, respectively. This scale measures state and trait anxiety and therefore gives a general anxiety score. An example item from the trait version is, “I feel inadequate”. Higher scores indicate higher levels of anxiety. The trait and state subscales of this scale have demonstrated sound internal consistency and reliability in different populations, such as students, previously (trait subscale, αs ≥ 0.86, state subscale, αs ≥ 0.83; Gaudry et al., 1975; Spielberger et al., 1983; Barnes et al., 2002).

Anxiety Sensitivity Index (ASI; Reiss et al., 1986). The ASI is a self-report questionnaire measuring fear of arousal-related sensations. Participants rated their agreement with 16 statements on a 5-point Likert scale from 0 *(Very little)* to 4 *(Very much)*, with higher scores demonstrating increased anxiety sensitivity. An example item is, “It is important to me to stay in control of my emotions.” This scale has demonstrated sound internal consistency and reliability previously (α = 0.88; Peterson and Heilbronner, 1987).

Depression Anxiety Stress Scale-21 (DASS-21; Lovibond and Lovibond, 1995). The DASS-21 is a self-report questionnaire with three 7-item subscales: Depression, Anxiety and Stress. Participants rated their agreement with the 21 statements on a 4-point Likert scale from 0 *(Did not apply to me at all)* to 3 *(Applied to me very much, or most of the time)*. Total scores for each subscale are multiplied by two to calculate the final score for each scale (Lovibond and Lovibond, 1995). Higher scores indicate greater depression, anxiety, or stress according to the subscale the score relates to. An example item is “I found it difficult to relax.” Subscales of this questionnaire have shown sound internal consistency and reliability in non-clinical samples in previous research (depression subscale, α = 0.94, anxiety subscale, α = 87, stress subscale, α = 0.91; Antony et al., 1998).

Beck Depression Inventory-II (BDI-II; Beck et al., 1996). The BDI-II is a self-report questionnaire consisting of 21 items rated on a 4-point Likert scale from 0 to 3, where a higher score represents increased depression symptoms. It measures mood, affect and physical activity over the past 2 weeks. For this measure, item 9, “Suicidal Thoughts or Wishes” was removed as a requirement from the Human Research Ethics Committee of Murdoch University. This scale has shown excellent previous internal consistency and reliability when testing university students (α = 0.93; Beck et al., 1996).

Positive and Negative Affect Schedule-X (PANAS-X; Watson and Clark, 1994). The PANAS is a self-report questionnaire with two 10-item subscales measuring positive and negative affect. Participants rated their agreement with the 20 statements on a 5-point Likert scale from 1 *(Very slightly)* to 5 *(Extremely)*. An adapted version of this measure was used, with 10 items measuring negative affect (four items from the Sadness, and six from the General Negative Affect subscale) and 10 items measuring positive affect (four items from the Joviality, and six from the General Positive Affect subscale) (Church et al., 2014). An example descriptor of feelings would be the degree to which one felt “afraid.” Higher scores on both subscales represented higher negative and positive affect, respectively. The adapted, and original version of this scale has shown sound previous internal consistency (positive affect subscale, αs ≥ 0.77, negative affect subscale, αs ≥ 0.80; Watson and Clark, 1994; Church et al., 2014).

### Procedure

The questionnaires were completed online via Qualtrics as part of pre-screening for another study. To allow participants to meet the time requirements needed to gain credit for completing these surveys, participants also completed surveys which were not the main focus of the present study. The questionnaires were presented in a random order for each participant. Students were recruited for this study through the Murdoch University research participant database and flyers were posted around the university. Wider community members were recruited via Social Media, such as through Facebook pages. Completion of the survey took approximately 30 min. Consent was given before completion of the questionnaires.

### Data Analysis

Due to recent research indicating that the Cronbach’s alpha (α) is a suboptimal measure of reliability, McDonald’s omega (ω) values are reported to indicate scale reliability (Hayes and Coutts, 2020), in addition to alphas to allow comparison with previous studies. McDonald’s omega values (ML) were calculated in SPSS (Version 24; IBM SPSS) using the plugin as described in Hayes and Coutts (2020).

AMOS (Version 24; IBM SPSS) was used to estimate all confirmatory factor analytic (CFA) models. The Maximum Likelihood estimator, which is the default in AMOS, was used for all CFA models. The goodness of fit of the CFA was evaluated with the chi-square goodness-of-fit tests, the root-mean-square error of approximation (RMSEA), the comparative fit index (CFI), goodness-of-fit index (GFI) and tucker-lewis index (TLI). Due to the sensitivity of the chi-square goodness-of-fit test to large sample sizes (Kline, 1998; Hu and Bentler, 1999; Kahn, 2006) the additional fit indices were also used to determine the fit of the models. In the present study, GFI, CFI, and TLI values of 0.90 and above were considered to reflect adequate fit, while values of 0.95 and above represented excellent fit (Knight et al., 1994; Kline, 1998; Hu and Bentler, 1999; Hooper et al., 2008). RMSEA values of 0.06–0.08 represented acceptable fit (Browne and Cudeck, 1993). For all models examined, modification indices for the covariances between error terms on items associated with each factor could correlate, as factors measured (state, trait, cognitive, and somatic anxiety) share strong theoretical associations (Cole et al., 2007; Hooper et al., 2008; Styck et al., 2020).

To examine the measurement invariance of the best fitting model across male and female participants, multigroup confirmatory factor analytic models were examined according to recommendations outlined by Brown (2006). Four levels of measurement invariance were tested sequentially (configural, weak, strong, and strict invariance). At each level, additional equality constrains were imposed, with the least strict of these being configural and the strictest, strict invariance. Configural invariance tests whether the same pattern of free and fixed loading occurs across genders. Weak configural invariance is established when factor loadings are constrained across groups. Strong invariance is confirmed through constraining item intercepts, in addition to factor loadings. The strictest form of measurement invariance is established when the sum of specific and error variance is the same across groups, in addition to factor loadings and item intercepts (Putnick and Bornstein, 2016). Given the sensitivity of chi-square tests to large sample sizes, other measures of fit were used to assess measurement invariance in addition to chi-square (Putnick and Bornstein, 2016). Therefore, to assess the four levels of measurement invariance the difference (Δ) in CFI and RMSEA were also examined (Chen, 2007). According to this, a change in CFI ≤ −0.010 and RMSEA ≤ −0.015 between sequential models was considered evidence for invariance.

To investigate concurrent and divergent validity of test scores, Pearson correlations were calculated between scores on the STICSA and scores on the other measures included. According to Cohen’s (1988) descriptors, correlations were examined according to: strong ± 0.5; moderate ± 0.30; weak ± 0.10. Due to the large number of correlations compared investigating concurrent and divergent validity, a new significance of *p* < 0.0006 from the significance level of *p* < 0.05, was used, as 72 multiple comparisons for each gender were conducted. For between-subjects *t*-tests comparing negative emotionality between genders, a new critical value of *p* < 0.003 was used, as 15 multiple comparisons were conducted. Steiger’s (1980) *Z* tests were also used to compare the concurrent and divergent validity of the STICSA and STAI with measures of interest. Many correlations were compared, and so a Bonferroni correction was applied while examining concurrent validity, resulting in a new critical value of *p* < 0.004. This was determined according to 12 multiple comparisons examining concurrent validity for each gender. For divergent validity with depression measures, a new critical value of *p* < 0.004 was applied according to 12 multiple comparisons over each gender. Comparison between the STICSA and STAI in terms of divergent validity, with measures of positive affect, negative affect and stress were also examined. To correct for six comparisons across each gender, a new critical value of *p* < 0.008 was used for these comparisons.

## Results

Little’s (1988) MCAR test was significant for scores on the negative subscale of the PANAS in male participants, however, missing values consisted of <5% of the total sample, therefore missing values were imputed using Expectation Maximization (Field, 2009). A *z* of ±3.29 was used for assessing univariate outliers (Field, 2009) for male and female responses separately. Therefore, responses from four male participants and 20 female participants were removed. Multivariate outliers were also assessed concurrently using Mahalanobis distance and removed according to a significance value of *p* < 0.001. According to this, two male participants and seven female participants were also removed. The final sample included in analyses was therefore 1,064 (*N* = 209 males, 855 females) participants.

Data were not normally distributed, according to ±1 skew and kurtosis (Morgan et al., 2001) with most scales for both males and females (skew: −0.31 – 1.50; kurtosis: −1.01 – 1.82) tending to be mildly positively skewed. Subsequently, scores on the ASI and somatic subscale of the STICSA state were transformed using the square root and inverse transformation, respectively. Sample size was considered large for female participants, and so normality could be assumed for measures answered by this sample (Ghasemi and Zahediasl, 2012). Therefore, skew and kurtosis were corrected according to responses made by male participants, with scores from females on these scales transformed for comparison. For clarity, descriptive statistics, and the direction of correlations reported, are according to those prior to transformation.

### Factor Structure of the STICSA

The two-factor state-trait correlated model, as seen in Supplementary Figure 2, had an inadequate fit (see Model 1 in Table 1). The two-factor cognitive-somatic correlated model, as seen in Supplementary Figure 3, also had an inadequate fit (see Model 2 in Table 1). In Model 1 RMSEA was higher than recommended and the CFI, TLI, and GFI were lower than the recommended cut offs. Further, although Model 2 according to RMSEA did have an acceptable fit, CFI, TLI, and GFI were lower than the recommended cut offs. The four-factor state-trait cognitive-somatic anxiety correlated model, as seen in Supplementary Figure 4, and hierarchical model, presented in Supplementary Figure 5, also had an inadequate fit (see Models 3 and 4 in Table 1, respectively). Both models had higher RMSEA than recommended and substantially lower CFI, TLI, and GFI than the recommended cut offs.

**TABLE 1:** Fit indices for STICSA models.

Model	χ ^2^	*df*	*p*	RMSEA	95% CI	CFI	GFI	TLI
(1) STICSA: two factor (state and trait)^b^	8953.69	794	<0.001	0.098	0.096–0.100	0.679	0.689	0.652
(2) STICSA: two factors (somatic and cognitive)^b^	3651.40	775	<0.001	0.059	0.057–0.061	0.887	0.809	0.874
(3) STICSA: four factors^b^	7561.53	806	<0.001	0.089	0.087–0.091	0.734	0.755	0.716
(4) STICSA: hierarchical^c^	8281.73	808	<0.001	0.093	0.091–0.095	0.706	0.741	0.687
(5) STICSA state: two factors (somatic and cognitive)	841.24	184	<0.001	0.058	0.054–0.062	0.930	0.929	0.920
STICSA trait: two factors (somatic and cognitive)^a^	647.48	185	<0.001	0.048	0.044–0.053	0.948	0.944	0.941

**CI, confidence interval. ^a^Model proposed by Ree et al. (2008). ^*b*^Models proposed by Grös et al. (2007). ^c^Model proposed by Roberts et al. (2016)*.*

The two-factor state and trait model separated into cognitive and somatic anxiety, as seen in Supplementary Figure 1, fit the data well (see Model 5 in Table 1). The RMSEA for the two parts of this model were <0.06. Further, CFIs were >0.93 and TFIs and GFIs were >0.92. According to all fit indices, model fit was achieved between the sample data and the model.

Standardized factor loadings for Model 5 were all significant (*p* < 0.001) and ranged from 0.46 to 0.79 (see Table 2). The fit indices of this model were superior to the four other alternative models examined.

**TABLE 2:** Factor loadings for the STICSA state: two factors (somatic and cognitive) and STICSA trait: two factors (somatic and cognitive) model.

	Standardized *(Unstandardized)* factor loadings			
	State		Trait	
Item number	State somatic	State cognitive	Trait somatic	Trait cognitive
1	0.56 (1.00)		0.56 *(1.00)*	
2	0.61 *(1.32)*		0.56 *(1.16)*	
6	0.56 *(0.76)*		0.54 *(0.94)*	
7	0.65 *(1.24)*		0.61 *(1.17)*	
8	0.65 *(1.01)*		0.66 *(1.20)*	
12	0.54 *(0.91)*		0.59 *(1.15)*	
14	0.64 *(1.24)*		0.59 *(1.06)*	
15	0.52 *(0.98)*		0.60 *(1.12)*	
18	0.70 *(1.04)*		0.71 *(1.21)*	
20	0.63 *(1.08)*		0.62 *(1.18)*	
21	0.56 *(0.99)*		0.59 *(1.15)*	
3		0.75 *(1.00)*		0.72 *(1.00)*
4		0.66 *(0.91)*		0.59 *(0.86)*
5		0.61 *(0.84)*		0.58 *(0.87)*
9		0.68 *(0.88)*		0.68 *(0.96)*
10		0.76 *(1.14)*		0.79 *(1.23)*
11		0.54 *(0.77)*		0.46 *(0.70)*
13		0.70 *(0.91)*		0.72 *(1.04)*
16		0.62 *(0.89)*		0.60 *(0.93)*
17		0.74 *(1.09)*		0.76 *(1.16)*
19		0.76 *(1.12)*		0.79 *(1.26)*
Factor correlations		*r*	*SE*	*p*
State somatic-state cognitive		0.70 *(0.18)*	*(0.01)*	<0.001
Trait somatic-trait cognitive		0.69 *(0.17)*	*(0.01)*	<0.001

### Multigroup Factor Structure

Comparison between models indicated that the two-factor, state-trait cognitive-somatic model (Model 5) was the best fitting model of the sample data. Therefore, to examine the fit of this model between male and female participants, measurement invariance of this model was examined. First, the fit of the baseline model was measured separately for male and female participants. Next, levels of measurement invariance were sequentially tested (configural, weak, strong, and strict) using further restrictive models. Results of these are reported in Table 3.

**TABLE 3:** Measurement invariance across male and female samples.

Model	χ ^2^	*df*	Δ *df*	TLI	CFI	Δ CFI	RMSEA	90% CI	Δ RMSEA
State									
Single-group									
Male (*n* = 209)	319.01*	184		0.916	0.927		0.059	0.048–0.070	
Female (*n* = 855)	789.50*	184		0.909	0.920		0.062	0.058–0.067	
Configural	1108.88*	368		0.910	0.921		0.044	0.041–0.046	
Weak	1139.06*	387	19	0.914	0.920	−0.001	0.043	0.040–0.046	0.000
Strong	1140.58*	390	3	0.914	0.920	0.000	0.043	0.040–0.045	0.000
Strict	1217.17*	415	25	0.914	0.915	−0.005	0.043	0.040–0.045	0.000
Trait									
Single-group									
Male (*n* = 209)	311.32*	185		0.917	0.927		0.057	0.046–0.068	
Female (*n* = 855)	593.02*	185		0.935	0.943		0.051	0.046–0.055	
Configural	904.83*	370		0.932	0.940		0.037	0.034–0.040	
Weak	923.44*	389	19	0.935	0.940	0.000	0.036	0.033–0.039	0.000
Strong	924.36*	392	3	0.936	0.940	0.000	0.036	0.033–0.039	0.000
Strict	995.05*	416	24	0.934	0.935	−0.005	0.036	0.033–0.039	0.000

*p < 0.001.

As can be seen in Table 3, configural, weak, strong, and strict measurement invariance was demonstrated for both male and female groups. In all instances, differences in CFI and RMSEA for all sequential models were less than thresholds used for assessing measurement invariance. Although chi-square difference tests were significant for levels of measurement invariance, like said previously, this statistic is overly sensitive to large sample sizes (Putnick and Bornstein, 2016). Therefore, for both male and female participants according to the two-factor state-trait cognitive-somatic model, measurement invariance between these samples was established.

### Descriptive Statistics and Internal Consistency

Descriptive statistics and internal consistencies for the measures administrated, collapsed across gender, as well as separated by gender, were calculated. Research indicates that women experience negative emotionality, such as anxiety, more than men. Further, women also experience different types of anxiety symptoms. Therefore, scores for males and females according to emotionality measures were compared via between-subjects *t-*tests. Associated results are shown in Table 4.

**TABLE 4:** Descriptive statistics and internal consistencies for measures collapsed across gender, as well as separated by gender, and between-subjects *t*-tests examining gender differences.

		Total			Male			Female		*t-test*	
Measure	*M (SD)*	α	ω	*M (SD)*	α	ω	*M (SD)*	α	ω	*t*(1062)	*d*
STICSA state	34.25 (10.51)	0.92	0.92	33.42 (10.16)	0.92	0.92	34.45 (10.60)	0.92	0.92	1.26	0.10
STICSA state somatic	15.20 (4.79)	0.86	0.86	14.83 (4.64)	0.87	0.87	15.29 (4.83)	0.86	0.86	1.46	0.10
STICSA state cognitive	19.05 (6.87)	0.90	0.90	18.59 (6.66)	0.90	0.90	19.16 (6.92)	0.90	0.90	1.08	0.08
STICSA trait	38.84 (10.67)	0.92	0.92	37.18 (10.20)	0.91	0.92	39.25 (10.75)	0.92	0.92	2.52	0.20
STICSA trait somatic	17.80 (5.29)	0.86	0.87	16.74 (5.00)	0.87	0.87	18.06 (5.33)	0.86	0.86	3.24*	0.26
STICSA trait cognitive	21.04 (6.56)	0.89	0.89	20.43 (6.40)	0.89	0.89	21.19 (6.59)	0.89	0.90	1.50	0.12
STAI trait	45.78 (10.92)	0.93	0.93	45.11 (10.49)	0.92	0.92	45.95 (11.03)	0.93	0.93	0.99	0.08
STAI state	40.89 (11.75)	0.94	0.94	40.21 (11.38)	0.94	0.94	41.05 (11.84)	0.94	0.94	0.93	0.07
ASI	22.93 (11.96)	0.90	0.90	20.90 (10.53)	0.87	0.87	23.42 (12.24)	0.90	0.91	2.46	0.31
DASS anxiety	9.57 (8.46)	0.82	0.83	7.96 (7.11)	0.78	0.78	9.96 (8.71)	0.83	0.83	3.48*^†^	0.25
DASS depression	11.14 (9.66)	0.89	0.89	12.00 (10.00)	0.89	0.90	10.93 (9.57)	0.89	0.89	1.44	0.11
BDI	13.82 (9.63)	0.90	0.90	12.82 (9.50)	0.90	0.90	14.06 (9.65)	0.90	0.90	1.67	0.13
PANAS positive	32.77 (7.43)	0.90	0.90	32.42 (7.53)	0.90	0.90	32.85 (7.41)	0.91	0.91	0.76	0.06
PANAS negative	22.00 (7.49)	0.89	0.89	22.00 (7.39)	0.88	0.88	22.00 (7.51)	0.89	0.89	0.00	0.00
DASS stress	14.53 (9.41)	0.85	0.85	12.67 (9.01)	0.84	0.84	14.99 (9.45)	0.85	0.86	3.21*	0.25

^†^df = 376.37, *p < 0.003.

As can be seen in Table 4, for both versions of the STICSA, in male and female samples, internal consistencies were good (αs > 0.91, ωs > 0.92). Internal consistencies on both versions of the STICSA for cognitive and somatic subscales across male and female samples were also acceptable (αs > 0.86, ωs > 0.86).

Additionally, across male and female samples, both subscales strongly correlated with each other on the state (*r*_*s*_ > 0.63, *p*s < 0.0001) and trait versions of the STICSA (*r*_*s*_ > 0.59, *p*s < 0.0001). The total scores of both versions of the STICSA across male and female samples were also strongly correlated (*r*_*s*_ > 0.78, *p*s < 0.0001), as were scores on the cognitive subscales and somatic subscales, across both versions, respectively *(r*_*s*_ > 0.63, *p*s < 0.0001).

### Gender Differences in STICSA and STAI

According to Table 4, female participants had significantly higher somatic trait anxiety as measured by the STICSA trait. The STAI, however, was not sensitive to this difference in trait anxiety between male and female participants. Further, women also had significantly higher anxiety and stress as measured by the anxiety and stress subscales of the DASS, respectively.

### Concurrent Validity

Correlations for measures administered to examine concurrent validity, separated by gender, are presented in Table 5. According to hypothesis one, it was predicted that the STICSA and its subscales would be more strongly correlated with measures of anxiety as compared to the STAI. To compare correlations between the STICSA and STAI with measures of anxiety, Steiger’s *Z*-values were calculated. Comparisons were conducted by comparing STICSA state scores with the STAI state, and STICSA trait scores with scores on the STAI trait. Whether Steiger’s *Z*-values were significantly different between the STICSA and STAI, are also indicated within Table 5. For actual Steiger’s *Z*-values, see Supplementary Table 1.

**TABLE 5:** Pearson correlations and Steiger’s *Z*, investigating the concurrent validity of the STICSA and STAI with measures of interest.

	ASI		DASS anxiety		STAI trait		STAI state	
	Male	Female	Male	Female	Male	Female	Male	Female
STICSA state	0.44*^a^	0.54*^a^	0.70*^a^	0.69*^a^	0.62*	0.68*	0.62*	0.67*
STICSA state somatic	0.39*	0.44*	0.66*^a^	0.62*^a^	0.46*	0.47*	0.53*	0.55*
STICSA state cognitive	0.41*	0.53*^a^	0.60*	0.62*^a^	0.66*	0.74*	0.61*	0.67*
STICSA trait	0.51*	0.59*^a^	0.74*^a^	0.73*^a^	0.68*	0.74*	0.59*	0.62*
STICSA trait somatic	0.45*	0.50*	0.76*^a^	0.68*	0.45*	0.51*	0.45*	0.49*
STICSA trait cognitive	0.46*	0.56*	0.58*	0.64*	0.73*	0.80*	0.59*	0.62*
STAI trait	0.37*	0.51*	0.54*	0.63*	–	–	0.77*	0.75*
STAI state	0.27*	0.40*	0.49*	0.53*	0.77*	0.75*	–	–

*^a^Significantly stronger correlation with the STICSA according to Steiger Z scores.*

As can be seen in Table 5, for both male and female participants, the total scores, and subscales of the STICSA state and trait were either moderately or strongly correlated with all anxiety measures.

#### STICSA vs. STAI

According to values in Table 5, the STICSA state and trait were significantly more correlated with both anxiety measures than the STAI state and trait, respectively, for female participants. For male participants, the STICSA state was significantly more correlated with both anxiety measures than the STAI state, while the STICSA trait was significantly more correlated with the anxiety subscale of the DASS as compared to the STAI trait. The cognitive subscale of the STICSA state was also significantly more associated with both anxiety measures than the STAI state for female participants. For male participants, the somatic subscale of the STICSA trait had a significantly stronger relationship with the anxiety subscale of the DASS as compared to STAI trait. Although non-significant for the remainder, the anxiety measures in most instances were more correlated with the STICSA for both genders as compared to the STAI.

### Divergent Validity

Correlations for measures administered to examine divergent validity, separated by gender, are presented in Table 6. According to hypothesis two, it was predicted that the STICSA as compared to the STAI would be less correlated with measures of depression, stress, negative and positive affect. Steiger’s *Z*-values were created to compare correlations between the STICSA and STAI with measures of divergent validity. Whether Steiger’s *Z*-values were significantly different between the STAI and STICSA, are also indicated in Table 6. For actual Steiger’s *Z*-values see Supplementary Table 2. Like with examination of concurrent validity, comparisons for divergent validity were done through comparing STICSA state scores with the STAI state, and STICSA trait scores with the STAI trait.

**TABLE 6:** Pearson correlations investigating the divergent validity of the STICSA and STAI with measures of interest.

	PANAS negative		PANAS positive		DASS stress		DASS depression		BDI-II	
Measure	Male	Female	Male	Female	Male	Female	Male	Female	Male	Female
STICSA state	0.61*	0.63*	−0.24*^a^	−0.38*^a^	0.54*	0.64*	0.55*	0.63*	0.62*	0.68*
STICSA state somatic	0.46*	0.47*^a^	−0.13^a^	−0.26*^a^	0.44*	0.52*^a^	0.36*^a^	0.45*^a^	0.49*^a^	0.51*^a^
STICSA state cognitive	0.62*	0.66*	−0.30*^a^	−0.41*^a^	0.54*	0.64*	0.59*	0.66*	0.62*	0.71*
STICSA trait	0.69*	0.70*^a^	−0.28*^a^	−0.39*^a^	0.67*	0.68*	0.58*^a^	0.65*^a^	0.68*	0.69*^a^
STICSA trait somatic	0.49*^a^	0.53*^a^	−0.10^a^	−0.24*^a^	0.54*	0.55*^a^	0.37*^a^	0.48*^a^	0.50*^a^	0.52*^a^
STICSA trait cognitive	0.71*	0.71*^a^	−0.36*^a^	−0.44*^a^	0.64*	0.67*	0.63*	0.67*^a^	0.69*	0.70*^a^
STAI trait	0.74*	0.75*	−0.60*	−0.65*	0.64*	0.66*	0.70*	0.73*	0.74*	0.77*
STAI state	0.60*	0.64*	−0.55*	−0.54*	0.56*	0.60*	0.54*	0.60*	0.64*	0.67*

^a^significantly stronger correlation with the STAI according to Steiger Z scores. *p < 0.0006.

#### Depression

As can be seen in Table 6, for both male and female samples, the total scores, and cognitive subscales of both the STICSA state and trait were strongly correlated with the depression subscale of the DASS and BDI-II. For both males and females, the somatic subscales of the STICSA state were also moderately correlated with the depression subscale of the DASS. While the somatic subscale of the STICSA trait, for both males and females were strongly correlated with the BDI-II. Further, according to Steiger *Z*-values, across genders, the somatic subscales of both the STICSA state and trait were significantly less correlated with both depression measures as compared to the cognitive subscales, *Z*s > −2.86, *ps* < 0.005.

#### Positive Affect

For male participants, the total score, and somatic subscales of the STICSA state and trait, were weakly correlated with a lack of positive affect. While the cognitive subscale of the STICSA state and trait for males was moderately correlated. For female participants, the total scores, and cognitive subscales of the STICSA state and trait, were moderately correlated with a lack of positive affect. While the somatic subscales of the STICSA state and trait for female participants, were only weakly correlated with a lack of positive affect.

#### Negative Affect

For male and female participants, the total scores, and cognitive subscales of the STICSA state and trait were strongly correlated with negative affect. In male participants, the somatic subscales were only moderately correlated with negative affect. For female participants, the somatic subscale of the STICSA state was also moderately correlated with negative affect, while the somatic subscale of the STICSA trait was strongly correlated.

### Stress

For both male and female participants, the total scores, and cognitive subscales of the STICSA state and trait were strongly correlated with the stress subscale of the DASS. While the somatic subscale of the STICSA state for males was moderately correlated with stress.

### STICSA vs. STAI

#### Depression

As can be seen in Table 6, for females, the total score of the STICSA trait and both subscales were significantly less correlated with the DASS depression and BDI-II as compared to STAI trait. The somatic subscale of the STICSA state was also significantly less correlated with both depression measures as compared to STAI state. Further, for male participants, the total score and somatic subscale of the STICSA trait was significantly less correlated with the DASS depression than the STAI trait. The somatic subscale of the STICSA trait was also significantly less correlated with the BDI-II than the STAI trait. No subscale of the STICSA for both male and female participants were significantly more correlated with either depression measure than the STAI.

#### Positive Affect

Both subscales of the STICSA state and trait for both male and female samples were significantly less correlated with positive affect as compared to the STAI state and trait.

#### Negative Affect

For female participants, the total score of the STICSA trait and both subscales of the STICSA trait were significantly less correlated with negative affect as compared to STAI trait. Further, the somatic subscale of the STICSA state was significantly less correlated with negative affect as compared to the STAI state in female participants. For male participants, the somatic subscale of the STICSA trait was significantly less correlated with negative affect than the STAI trait. Further, for the remainder, although non-significant, the STICSA in most cases was less correlated with negative affect than the STAI.

#### Stress

For female participants, the somatic subscale of the STICSA state and trait were significantly less correlated with stress as compared to STAI state and trait, respectively. Further, although non-significant, within the male sample, the STAI was more correlated with the stress subscale of the DASS than the STICSA in most cases.

## Discussion

The present study aimed to examine the factor structure of the STICSA and to compare the concurrent and divergent validity of the STICSA with the STAI, while separating between genders. The best fit of the sample data was the two-factor state-trait cognitive-somatic model, supporting the original factor structure put forward by Ree et al. (2008). Like Carlucci et al. (2018), the factor structure of this model was invariant between male and female participants. In most cases, for both men and women, the STICSA as compared to the STAI, either had a larger or significantly larger relationship with other anxiety measures. This finding mostly supported hypothesis one in relation to concurrent validity. In most cases, for both genders, the STICSA as compared to the STAI, also had either a weaker or significantly weaker relationship with depression, stress, negative and positive affect. Therefore, overall, the STICSA also had superior divergent validity than the STAI, supporting hypothesis two.

### Factor Models of the STICSA

The two-factor state-trait cognitive-somatic model was the best fit of the sample data, with this structure invariant between genders. This finding supports previous research (Ree et al., 2008; Carlucci et al., 2018; Styck et al., 2020) and indicates that the STICSA state and trait can be administered separately in research to assess dispositional (trait) and situational (state) cognitive and somatic anxiety. Our study also extends on these studies, as we are the first to find support for this factor structure in English-speaking male and female participants. The findings of the present research are in contrast with Roberts et al. (2016) who found support for the hierarchical model, and Grös et al. (2007), who found support for the four-factor model. Future research should endeavor to examine the factor structure of these models using samples specific to Roberts et al. (2016) and Grös et al. (2007). Although Roberts et al. (2016) examined the factor structure of the STICSA using the English version of this questionnaire, they indicated that their sample was largely heterogeneous. Therefore, a similar sample to Roberts et al. (2016) should be tested in future. Further, Grös et al. (2007) tested a clinical sample of participants. This indicates that the factor structure originally put forward by Ree et al. (2008), despite being supported and invariant between genders when using a non-clinical sample, may deviate in samples with clinical levels of anxiety.

### STICSA Versus STAI

#### Concurrent Validity

Results of this study also mostly support hypothesis one. The STICSA and its subscales were strongly to moderately correlated with all anxiety measures for both male and female participants. Interestingly, scores on the STICSA, its subscales and the STAI, were less correlated with the ASI in male participants as compared to females. This finding supports previous research indicating that anxiety sensitivity is not a strong indicator of an anxiety response in men (Deacon et al., 2003; Norr et al., 2015).

In addition to superior concurrent validity, in most cases, the total scores of the STICSA and somatic subscales had larger correlations with measures of anxiety as compared to the trait. Only the somatic subscale of the STICSA trait in females was less correlated with the ASI than the STAI trait. This difference in correlations, however, was minor and not statistically significant. Collectively, these findings add to research indicating that the STICSA somatic subscales and total scores, capture more unique variance related to anxiety via inclusion of items measuring somatic anxiety/tension (Clark and Watson, 1991; Grös et al., 2007, 2010; Roberts et al., 2016). In extension to prior findings, however, we also found that the cognitive subscales of the STICSA in most cases, were also more correlated with other anxiety measures as compared to the STAI. This indicates that even though both the STICSA and STAI include items capturing cognitive anxiety, the STICSA captures this facet to a greater degree. This finding could be attributed to the STAI, in addition to capturing negative affect, which is related to cognitive anxiety (Grös et al., 2007), also capturing variance related to a lack of positive affect.

#### Divergent Validity

Hypothesis two was also mostly supported by the results of this study. Although most correlations between the STICSA and measures of depression were moderate to strong, the STICSA in most cases, was either less correlated or significantly less correlated with these measures than the STAI. Also supporting previous research and consistent with the tripartite model (Clark and Watson, 1991; Roberts et al., 2016), the somatic subscales of the STICSA were significantly less correlated with depression than the cognitive subscales. These findings support previous research indicating that the STICSA, especially the somatic anxiety subscales, are less correlated with depression (Grös et al., 2007, 2010; Roberts et al., 2016). The somatic subscales of the STICSA in male and female participants, were also non-significantly and weakly correlated with positive affect, respectively. Further, total and subscale scores on the STICSA in both samples, were either less correlated or significantly less correlated with positive affect than the STAI. This finding corroborates our findings related to depression, and previous research (Grös et al., 2007, 2010; Roberts et al., 2016) indicating that the STICSA is less correlated with depression, as a lack of positive affect is specific to depression and not anxiety (Caci et al., 2003).

In light of our findings, future studies should seek to examine whether the STICSA and its subscales are less correlated with positive affect and depression within clinical samples. While also examining whether these correlations are smaller than those observed with the STAI. This would extend on research conducted by Grös et al. (2007) who did not measure the STICSA in relation to positive affect and did not separate the STICSA according to its subscales. If the STICSA is found to be less correlated with a lack of positive affect and depression in future studies, the STICSA could be employed within a clinical setting to help minimize misdiagnosis between disorders. Use of this scale could subsequently improve the effectiveness of both therapeutic and pharmaceutical interventions (Ballenger, 2000; Tiller, 2013). For instance, the STICSA in addition to administering a measure of depression, might help distinguish between behavioral profiles of individuals with an anxiety disorder, depressive disorder or comorbid anxiety and depression diagnosis. It must be reiterated, however, that these recommendations could only be put into practice once the STICSA is validated within a clinical population.

In the study by Roberts et al. (2016), the STICSA trait and its subscales were similarly correlated with negative affect as compared to the STAI. In our study, in both men and women, the somatic subscale of the STICSA trait was significantly less correlated with negative affect than the STAI. This suggests, that although anxiety and depression share high levels of negative affect (Clark and Watson, 1991), the somatic subscale of the STICSA trait captures less variance related to negative affect, and more unique variance related to somatic anxiety/tension. This result has implications for future research within both non-clinical and clinical samples. Within the experimental setting, researchers have indicated that anxiety inductions frequently do not induce enough state anxiety needed to impair cognitive performance (Deffenbacher et al., 2004). Administering the STICSA trait version of the somatic subscale, however, prior to testing in future research, could ensure that only participants with a vulnerability to this type of induction are tested. Future research should also seek to replicate our findings within a clinical sample. Although anxiety disorders share variance with negative affect (Clark and Watson, 1991), certain disorders have a stronger relationship with somatic anxiety than others (den Hollander-Gijsman et al., 2010). For instance, although panic disorder and generalized anxiety disorder share variance with negative affect, panic disorder also includes variance specific to somatic anxiety (den Hollander-Gijsman et al., 2010). Therefore, future research should seek to examine whether the somatic subscales of the STICSA can aid differentiation between such disorders when using a clinical sample.

Within our study in both genders, the stress subscale of the DASS was less correlated with the somatic subscales of the STICSA as compared to the STAI. Although the comparison between stress and anxiety has been relatively overlooked by previous research (Roberts et al., 2016; Carlucci et al., 2018), our findings indicate that the STICSA somatic subscales share less common variance with stress. According to Lovibond and Lovibond (1995) the stress scale of the DASS captures variance specific to a persistent state of arousal with a lower threshold for becoming upset. Further, most overlap between the anxiety and stress subscales of the DASS were indicated between items measuring nervous tension and energy (Lovibond and Lovibond, 1995). Within both the STAI state and trait, for example, items are included that explicitly ask participants how tense, nervous, and jittery they are. No such items are contained within the STICSA somatic subscales. Distinguishing between stress and anxiety is essential, as the level of stress experienced by an individual differentiates adaptive coping strategies from those that are maladaptive (Lazarus and Folkman, 1984; Lazarus, 1993). Anxiety, however, although related to stress, has important clinical implications relevant to determining the risk of developing an anxiety disorder (Rose and Devine, 2014). Anxiety also helps identify how a person will act in a variety of situations due to indicating a psychological response which generalizes between situations (Sarason, 1984).

### Gender Differences in STICSA

Although the factor structure of the two-factor state-trait cognitive-somatic model of the STICSA was invariant between genders, differences in the magnitude of anxiety symptoms existed between males and female samples. Female participants in our study, had significantly higher somatic trait anxiety on the STICSA as compared to men (Balsamo et al., 2015). This supports previous research indicating that women within non-clinical samples experience physiological anxiety symptoms to a larger extent than men (Stewart et al., 1997). Further, in our study, women also had higher anxiety as measured by the anxiety subscale of the DASS, with this also supporting previous research (Bayram and Bilgel, 2008; Imam, 2008). No difference in cognitive trait anxiety as measured by the STICSA between men and women occurred. Taken together, these findings support the proposition that women not only experience anxiety to a greater magnitude than men, but also experience certain anxiety symptoms more frequently (McLean and Anderson, 2009; McLean et al., 2011). Further, women were also found to experience more stress than men, also supporting previous research (Bayram and Bilgel, 2008; Imam, 2008). Our study is the first to find a gender division when using the STICSA, and therefore extends on previous research. Separately, and of note, the distinction between males and females in term of trait anxiety, did not occur according to the STAI. This most likely occurred through omission of variance related to somatic anxiety within the STAI (Roberts et al., 2016). The STAI being unable to identify gender differences in anxiety responses highlights another limitation associated with use of this measure. Findings of our study, therefore, not only indicate that the STICSA is a purer measure of anxiety (Grös et al., 2007), but also indicates that the STICSA is more sensitive to gender differences.

### Limitations

Several limitations existed within this study. This study consisted of a non-clinical sample, which was predominately female and highly educated. Therefore, despite the findings of this study having implications for future research into clinical populations, the generalisability of our findings to this sample might be limited. Further, the Form-X state measure of the STAI (Spielberger et al., 1970) was used instead of the Form-Y. This version is suggested to be confounded by depression to a greater degree than the newer version (Spielberger et al., 1983). However, this limitation is more pronounced in the trait, rather than state version of the Form-X (Spielberger et al., 1983; Hishinuma et al., 2001). Further, according to Spielberger et al. (1983), both the Form-X and Y are highly correlated and can be considered essentially equivalent. Additionally, in the current study, the state version of the STAI was less correlated with depression than the STAI trait, suggesting minimal impact of using this version of the STAI state.

### Future Directions

In our study the two-factor cognitive-somatic state-trait model was the best fitting model of the STICSA, supporting the original structure proposed by Ree et al. (2008), the investigation by Carlucci et al. (2018) and Styck et al. (2020). Further, our study like Carlucci et al. (2018), found support for the invariance of this structure across genders. Other studies, however, in contrast with our findings and previous research, have found support for other models, namely the four-factor model and the hierarchical model (Grös et al., 2007; Roberts et al., 2016). As previously stated, demographic differences existed between the present study and that conducted by Grös et al. (2007) and Roberts et al. (2016). Future research should therefore aim to replicate the factor structure observed here using a range of populations while separating between male and female samples.

Like that said above, future research should seek to examine whether the STICSA and its subscales are less correlated with depression and a lack of positive affect within clinical settings. Further, examination of whether the somatic subscales are less correlated with negative affect is also warranted. The STICSA within these populations might help with diagnosis of certain disorders (den Hollander-Gijsman et al., 2010). Specifically, scores on the STICSA might allow for differentiating between depression, anxiety and comorbid anxiety and depression. While scores on the somatic subscale might help distinguish between individuals with different types of anxiety disorders (den Hollander-Gijsman et al., 2010). Further, use of the somatic subscale within the experimental setting with non-clinical populations could help determine anxiety vulnerability to certain anxiety inductions. As indicated by gender differences on the STICSA, future research should examine these avenues while separating between genders.

## Conclusion

This study extends on previous research into the reliability and validity of the STICSA. Our analysis conducted on the STICSA provides evidence that the cognitive and somatic anxiety items of the STICSA load separately onto state and trait anxiety within an English-speaking non-clinical population (Ree et al., 2008). Our findings also add to the literature as this factor structure was invariant between genders. This study extends support for the proposition that the STICSA is a purer measure of anxiety as compared to the STAI. The STICSA was found to be more correlated with various measures of anxiety as compared to the STAI, highlighting its superior concurrent validity. Further, the STICSA overall, was also found to be less strongly correlated with measures of depression, negative and positive affect, and stress. This highlights that scores on the STICSA, if further examined within a clinical sample, might allow for the differentiation of not only anxiety and depressive disorders, but also between different anxiety disorders, with this having potential implications for treatment.

## Data Availability Statement

The raw data supporting the conclusions of this article will be made available by the authors, without undue reservation.

## Ethics Statement

The studies involving human participants were reviewed and approved by Human Research Ethics Committee, Murdoch University. The patients/participants provided their informed consent to participate in this study.

## Author Contributions

IT collected, analyzed, and prepared the draft and final manuscript. GC and VL provided feedback on draft manuscripts to prepare it for publication. All authors contributed to the article and approved the submitted version.

## Conflict of Interest

The authors declare that the research was conducted in the absence of any commercial or financial relationships that could be construed as a potential conflict of interest.
