# Supplementary material for: Dimensionality and Measurement Invariance of the State-Trait Inventory for Cognitive and Somatic Anxiety (STICSA) and Validity Comparison With Measures of Negative Emotionality
Source: Front Psychol. 2021 Jun 7;12:644889. doi: 10.3389/fpsyg.2021.644889 (PMC8215200; doi:10.3389/fpsyg.2021.644889)
Supplement: Supplementary file 1 [file Data_Sheet_1.docx]

#
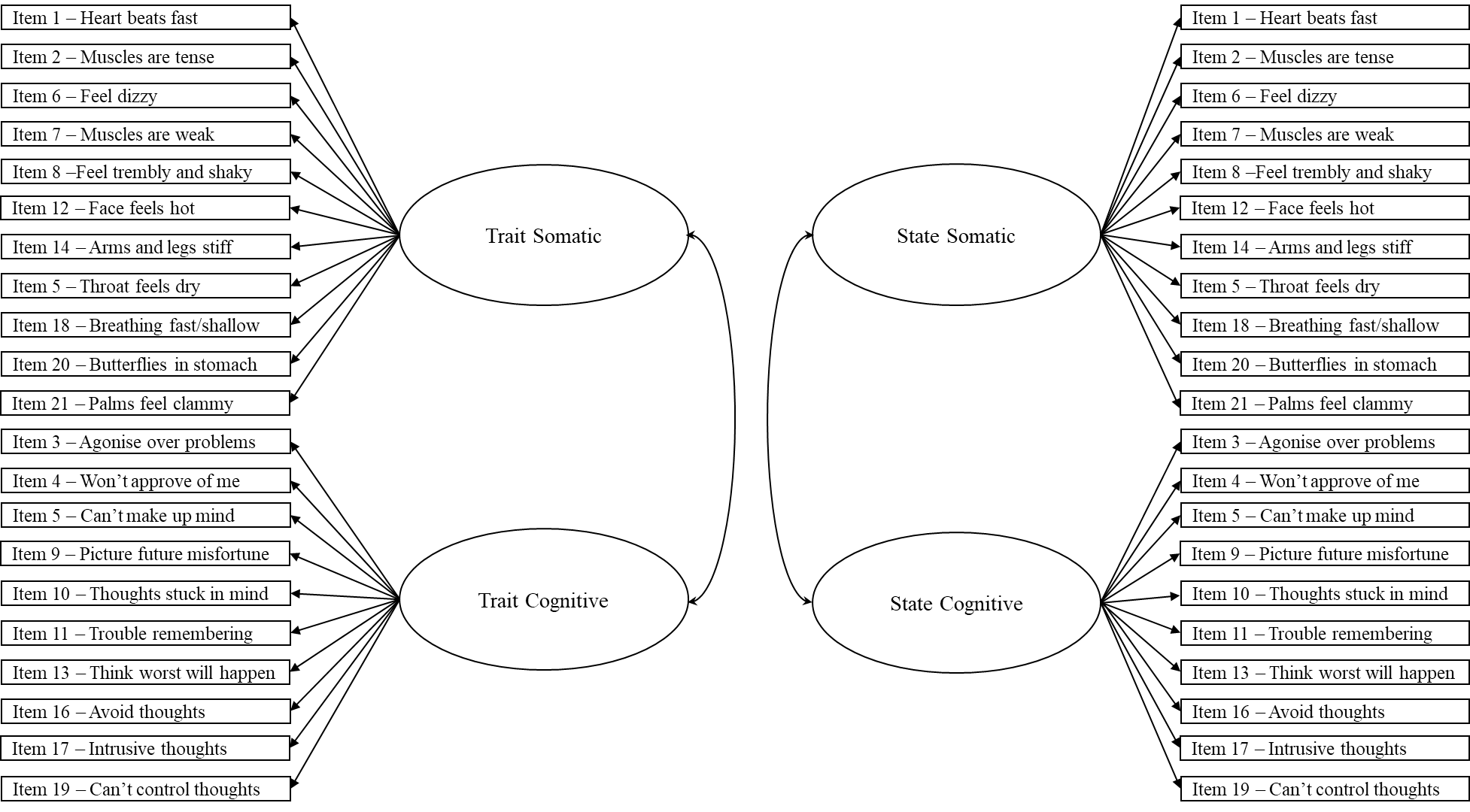
­Supplementary Materials

*
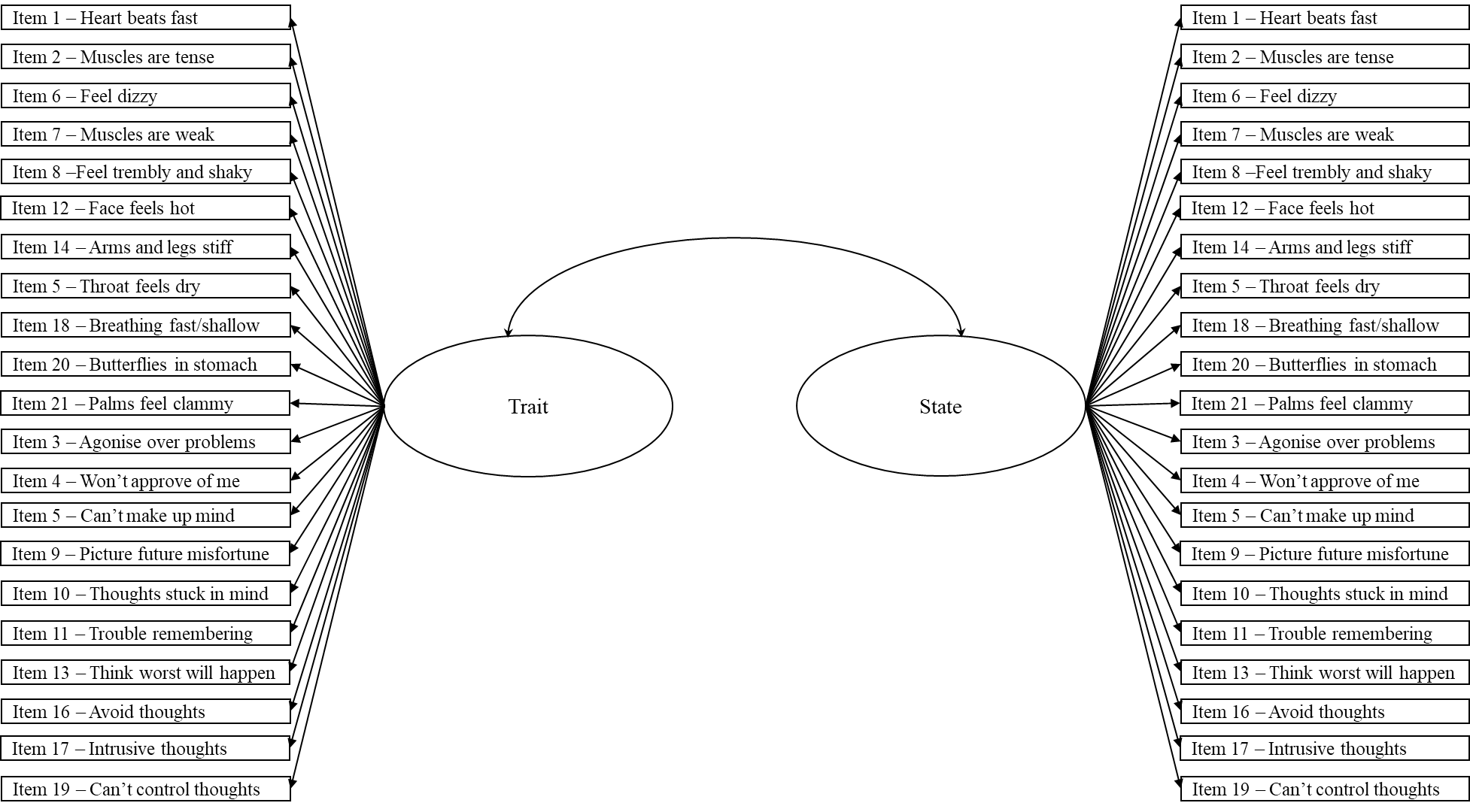
Figure S1.* Two-factor state and trait separated into cognitive and somatic anxiety model for the STICSA.

*Figure S2****.*** Two-factor state-trait correlated model of the STICSA.


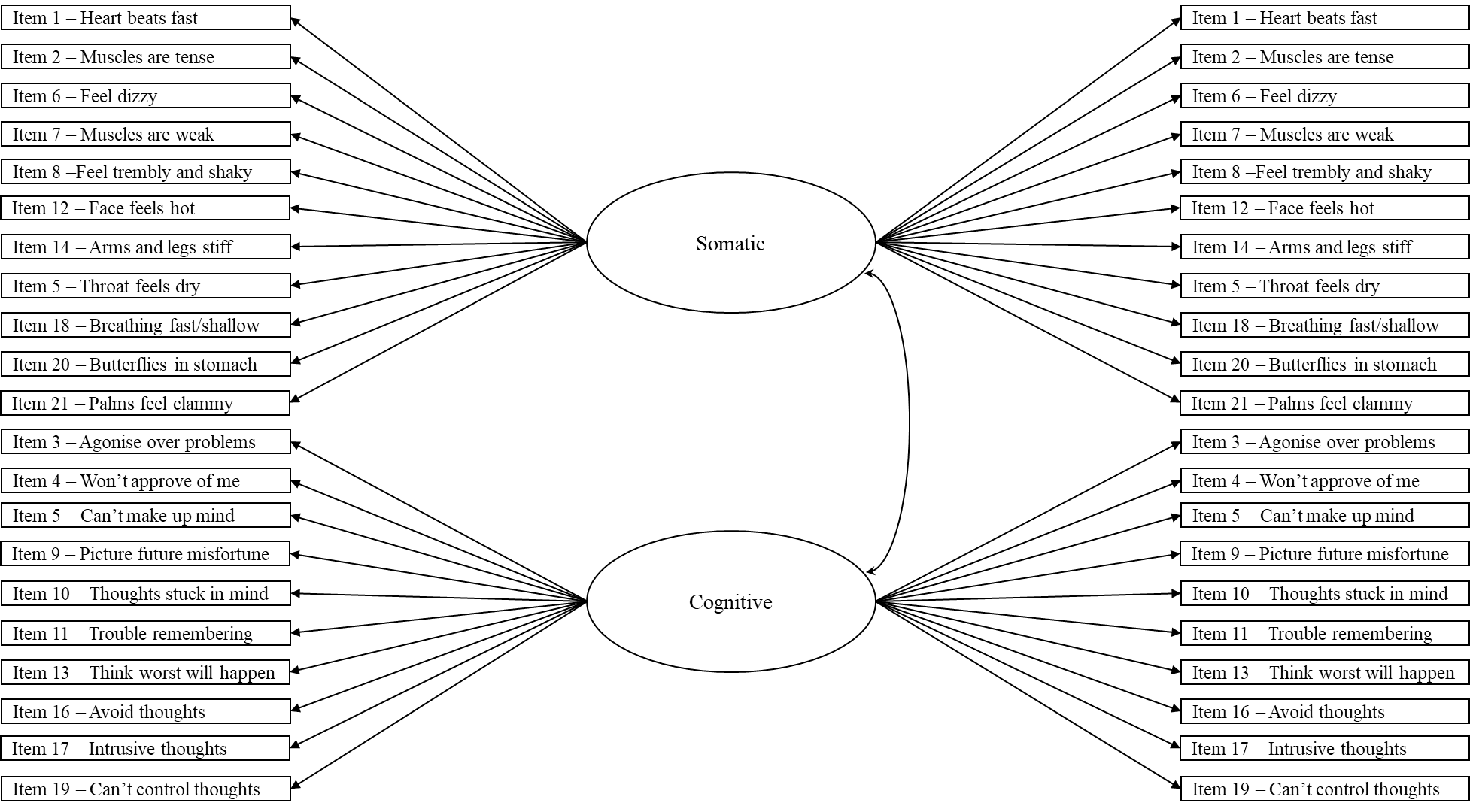
*Figure S3****.*** Two-factor cognitive-somatic correlated model of the STICSA.


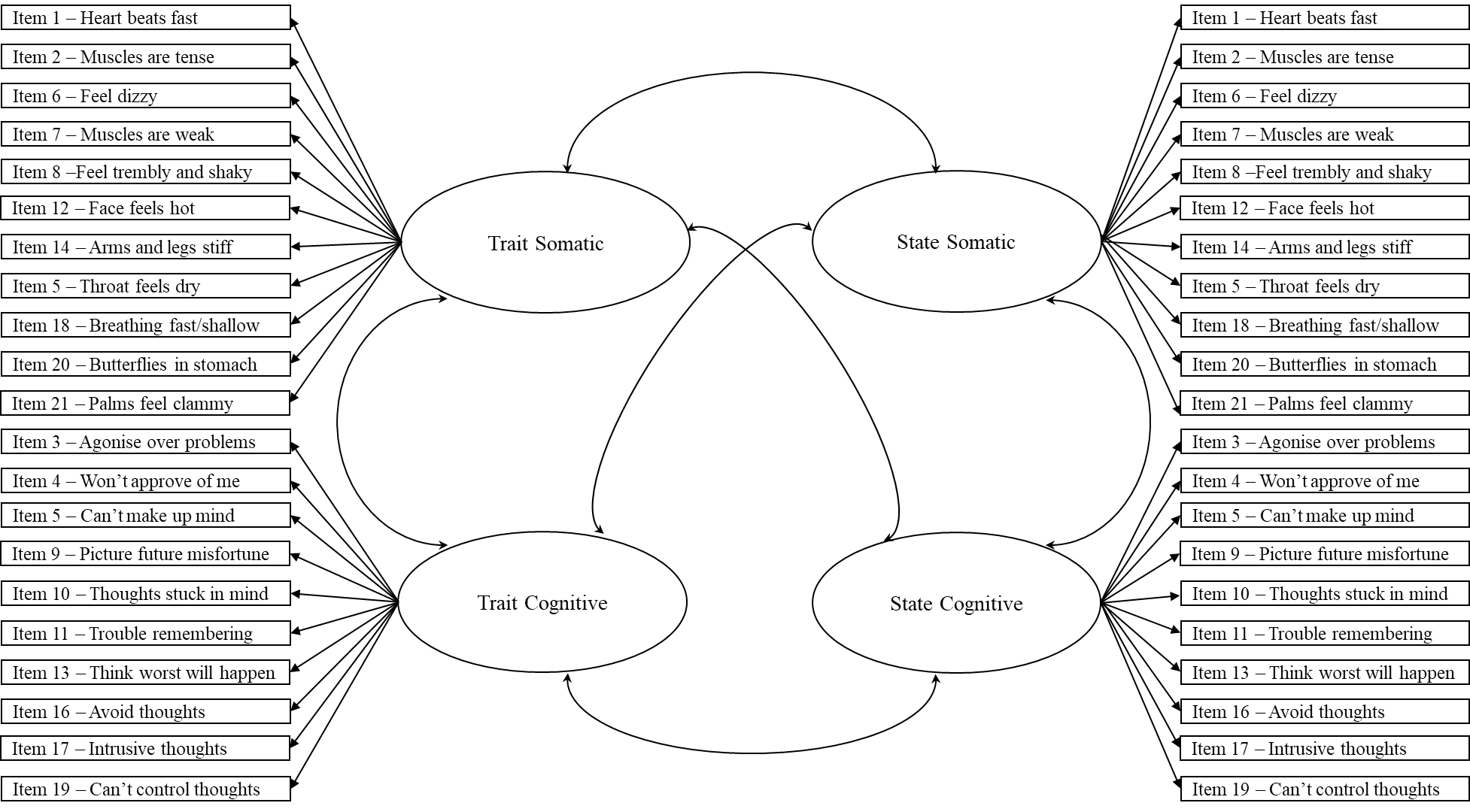


*Figure S4.* Four-factor state-trait cognitive-somatic anxiety model of the STICSA. Adapted from “Factor structure and validity of the State-Trait Inventory for Cognitive and Somatic Anxiety”, by Karen E. Roberts, Trevor A. Hart and John D. Eastwood, 2016, *Psychological Assessment*, p. 135. Copyright 2016 by American Psychological Association.

*
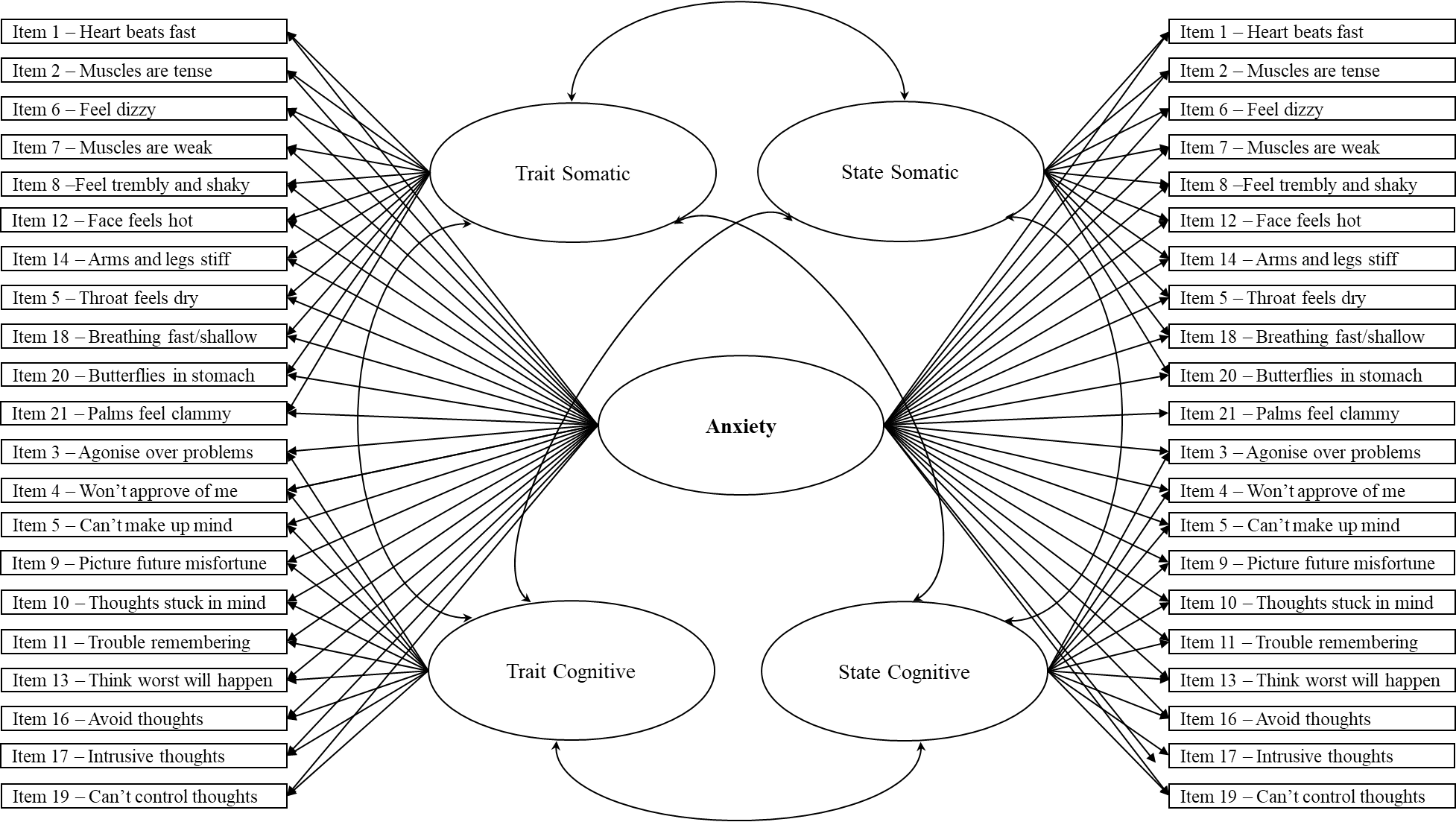
Figure S5.* Hierarchical model of the STICSA with a first order global anxiety factor and second order intercorrelated state-trait cognitive-somatic subdomains. Adapted from “Factor structure and validity of the State-Trait Inventory for Cognitive and Somatic Anxiety”, by Karen E. Roberts, Trevor A. Hart and John D. Eastwood, 2016, *Psychological Assessment*, p. 136. Copyright 2016 by American Psychological Association.

Table S1

*Comparison between the STICSA and STAI, in terms of their relationship with anxiety.*

|  | ASI | |  | DASS anxiety | |
| --- | --- | --- | --- | --- | --- |
| Comparison between measures | Male | Female |  | Male | Female |
| STICSA state vs. STAI state | 2.95^*a^ | 5.62*^a^ |  | 4.54*^a^ | 7.74*^a^ |
| STICSA state somatic vs. STAI state | 1.84 | 1.18 |  | 3.40*^a^ | 3.81*^a^ |
| STICSA state cognitive vs. STAI state | 2.34 | 5.28*^a^ |  | 2.13 | 4.38*^a^ |
| STICSA trait vs. STAI trait | 2.81 | 4.09*^a^ |  | 5.24*^a^ | 6.11*^a^ |
| STICSA trait somatic vs. STAI trait | 1.21 | -.36 |  | 4.73*^a^ | 2.35 |
| STICSA trait cognitive vs. STAI trait | 1.91 | 2.79 |  | 1.20 | .56 |

*Note.* ^a^ significantly stronger correlation with the STICSA according to Steiger Z.

* *p<*.004.

Table S2

*Comparison of the STICSA and the STAI with measures of depression, positive and negative affect, and stress.*

|  | DASS depression | |  | BDI-II | |  | PANAS positive | |  | PANAS negative | |  | DASS stress | |
| --- | --- | --- | --- | --- | --- | --- | --- | --- | --- | --- | --- | --- | --- | --- |
| Comparison between measures | Male | Female |  | Male | Female |  | Male | Female |  | Male | Female |  | Male | Female |
| STICSA state vs. STAI state | .10 | 1.16 |  | -.34 | .95 |  | 5.96**^b^ | 6.92**^b^ |  | .22 | -.30 |  | -.31 | 1.90 |
| STICSA state somatic vs. STAI state | -3.23*^b^ | -5.94*^b^ |  | -2.93*^b^ | -6.38*^b^ |  | 7.15**^b^ | 9.92**^b^ |  | -2.62 | -6.58**^b^ |  | -2.12 | -3.50**^b^ |
| STICSA state cognitive vs. STAI state | 1.00 | 2.86 |  | -.40 | 2.37 |  | 4.78**^b^ | 5.44**^b^ |  | .33 | .75 |  | -.51 | 1.70 |
| STICSA trait vs. STAI trait | -2.91*^b^ | -4.57*^b^ |  | -1.87 | -4.99*^b^ |  | 6.77**^b^ | 13.25**^b^ |  | -1.40 | -3.40**^b^ |  | .68 | 1.05 |
| STICSA trait somatic vs. STAI trait | -5.76*^b^ | -10.30*^b^ |  | -4.88*^b^ | -10.94*^b^ |  | 7.83**^b^ | 14.83**^b^ |  | -4.83**^b^ | -9.65**^b^ |  | -1.79 | -4.60**^b^ |
| STICSA trait cognitive vs. STAI trait | -1.83 | -3.55*^b^ |  | -1.64 | -4.52*^b^ |  | 5.52**^b^ | 12.11**^b^ |  | -.83 | -3.10**^b^ |  | -.03 | .19 |

*Note.* ^b^ significantly stronger correlation with the STAI according to Steiger Z.

* *p* < .004, ** *p <* .008.
